# Systematic Evaluation of IMU Sensors for Application in Smart Glove System for Remote Monitoring of Hand Differences

**DOI:** 10.3390/s25010002

**Published:** 2024-12-24

**Authors:** Amy Harrison, Andrea Jester, Surej Mouli, Antonio Fratini, Ali Jabran

**Affiliations:** 1 Engineering for Health Research Centre, Aston University, Aston Triangle, Birmingham B4 7ET, UK; 200290698@aston.ac.uk (A.H.); s.mouli@aston.ac.uk (S.M.); a.fratini@aston.ac.uk (A.F.); 2 Hand and Upper Limb Service, Birmingham Children’s Hospital, Birmingham B4 6NH, UK; andrea.jester@nhs.net

**Keywords:** smart glove, remote monitoring, hand differences, inertial measurement unit, hand function assessment, finger joint, range of motion, telehealth, wearable technology, upper limb

## Abstract

Human hands have over 20 degrees of freedom, enabled by a complex system of bones, muscles, and joints. Hand differences can significantly impair dexterity and independence in daily activities. Accurate assessment of hand function, particularly digit movement, is vital for effective intervention and rehabilitation. However, current clinical methods rely on subjective observations and limited tests. Smart gloves with inertial measurement unit (IMU) sensors have emerged as tools for capturing digit movements, yet their sensor accuracy remains underexplored. This study developed and validated an IMU-based smart glove system for measuring finger joint movements in individuals with hand differences. The glove measured 3D digit rotations and was evaluated against an industrial robotic arm. Tests included rotations around three axes at 1°, 10°, and 90°, simulating extension/flexion, supination/pronation, and abduction/adduction. The IMU sensors demonstrated high accuracy and reliability, with minimal systematic bias and strong positive correlations (*p* > 0.95 across all tests). Agreement matrices revealed high agreement (<1°) in 24 trials, moderate (1–10°) in 12 trials, and low (>10°) in only 4 trials. The Root Mean Square Error (RMSE) ranged from 1.357 to 5.262 for the 90° tests, 0.094 to 0.538 for the 10° tests, and 0.129 to 0.36 for the 1° tests. Likewise, mean absolute error (MAE) ranged from 0.967 to 4.679 for the 90° tests, 0.073 to 0.386 for the 10° tests, and 0.102 to 0.309 for the 1° tests. The sensor provided precise measurements of digit angles across 0–90° in multiple directions, enabling reliable clinical assessment, remote monitoring, and improved diagnosis, treatment, and rehabilitation for individuals with hand differences.

## 1. Introduction

Human hands are highly complex actuators, consisting of a total of 27 bones, 18 joints, and 39 muscles, which afford over 20 degrees of freedom (DOF) [1]. They allow humans to communicate, as well as explore and modify the environment. Hand function is required for many activities of daily living (ADLs) such as eating and self-care but also for participation in work and leisure activities. The role of the digits in hand function is especially critical, as they facilitate fine motor skills, object manipulation, and precision grip, all of which are key components of performing daily activities and complex tasks.

Hand differences, whether congenital or acquired, can lead to partial or total loss of hand function [2]. Congenital hand differences include syndactyly (fused digits) and polydactyly (extra digits) and can be due to developmental disorders such as cerebral palsy [3]. Hand differences can also be acquired through overuse, injury (e.g., hand fractures), or medical conditions such as stroke, arthritis, Parkinson’s disease, cancer, and diabetes mellitus [2]. Consequently, these hand differences can negatively impact patients’ quality of life and their ability to independently perform ADLs [2,4,5,6,7]. This can undermine not only the patients’ physical health but also their psychological, social, and economic well-being [2,8].

In many cases, surgical and non-surgical interventions are needed to treat and manage hand differences, as well as their underlying medical conditions [2]. Moreover, digit-specific rehabilitation or reconstructive techniques have been shown to restore function effectively in cases of trauma or degenerative diseases. The ability to both accurately and reliably measure hand function, particularly the digits, is fundamental for the evaluation of these interventions and successful rehabilitation [9]. However, currently, clinicians predominantly rely on their own observations to evaluate the effectiveness of these interventions, as well as to monitor the progression or regression of hand function over time. To this aim, several questionnaires, and assessment tests have been developed; however, these do not capture the complexity of hand movements in three-dimensional (3D) space. They are often limited to metrics such as the time required to complete a set of functional tasks and the assessors’ scores of task completion [10,11].

### Digit-Tracking Technological Solutions

The current technological solutions for tracking the digits can generally be grouped as either vision-based or sensor-based [12]. Vision-based solutions typically make use of RGB cameras; RGB-D (depth) cameras and infrared cameras have been demonstrated for a variety of applications [12,13]. For example, they have been shown to enable hand gesture detection, allowing deaf-mute participants to communicate and enabling clinicians to control medical devices, examine medical images, and interact with patients with minimal contact, thereby reducing the risk of cross-contamination [13,14,15]. In the automotive industry, vision-based gesture systems have been demonstrated to allow gesture-controlled interaction with a vehicle’s multimedia system, allowing drivers to focus on the road and potentially reducing the risk of accidents [15]. However, factors such as occlusion are well-documented issues with vision-based solutions, restricting their deployment to real-life applications. Hands need to be in the foreground, in view of the cameras to allow hand segmentation to work, which may not always be possible for complex hand movements performed by patients with hand differences [12,15,16,17]. Further, current vision-based solutions need to be trained on computer algorithms trained on images with simple, well-defined background environments, which can be challenging when deploying them into real life where the background is often complex, along with the simultaneous movement of all digits [15,18].

Addressing the limitations of vision-based solutions, the recent decade has witnessed a significant increase in interest in the development of sensor-based smart glove systems, typically based on Inertial Measurement Units (IMUs), flex sensors, and strain sensors [17]. IMU sensors are microelectromechanical systems (MEMS) that employ a combination of triaxial accelerometers, gyroscopes, and magnetometers to measure linear acceleration, angular velocity, and magnetic heading. They allow the capture of 3D movement data by converting inertial signals into precise positional and orientation outputs. Flex sensors use piezo-resistive elements that change their resistance as they are bent or flexed. For optimal performance, they need to be placed precisely at joint locations to allow an accurate one-to-one correspondence between joint bending and sensor reading [17]. This poses challenges when measuring finger abduction and adduction [17,19]. In addition to this, the continuous bending of the flex sensors causes them to break rapidly, leading to a short lifespan. Strain sensors rely on capacitive/resistance elements that change signals as they stretch.

Sensor-based glove systems have been used in many fields including extended reality, gaming, and entertainment, as well as training, simulation, and rehabilitation for capturing the 3D movements of hands over time [16,17]. Gloves have been developed for capturing digit movements and gestural analysis for the translation of sign language and the recreation of virtual characters (together with whole-body suits) for the film and television field [20,21]. When coupled with tactile information, smart gloves have been demonstrated to allow an accurate perception of the shapes and weights of virtual objects in a simulation environment, with potential applications in simulating real-world physiotherapy scenarios and clinical diagnoses [16,20,21].

The application of sensor-based to-hand differences will enable a more direct, quantitative comparison between interventions and monitoring of hand function. The data captured from these glove systems can allow for more informed clinical decisions and thus improve functional outcomes and quality of life. Further, they can enable remote monitoring of hand function, which can provide savings in clinic and clinician’s time, as well as the number of visits to specialists’ units.

Despite the recent development of sensor-based solutions, there is a lack of information on the glove sensors’ accuracy and reliability when measuring the motion range (joint rotations), especially in IMU-based gloves [17]. Systematic characterisation of the glove sensors’ accuracy and reliability in measuring the range of motions (ROMs) would help determine their tracking and positioning capabilities and thus their suitability for assessing hand differences.

We developed an IMU-based smart glove dedicated to application in hand differences that allowed for the measurement of 3D joint rotations at all five digits. The aim of this study was to systematically evaluate the accuracy and reliability of IMU sensors in measuring the 3D range of motion of the digits against an industrial robotic arm. To achieve this, 3D rotations of the glove sensor were performed, simulating the extension-flexion, supination-pronation, and abduction-adduction of digits.

## 2. Materials and Methods

### 2.1. Hardware Design

The key electronic architecture of the data acquisition glove comprised a bespoke printed circuit board (PCB), six microelectromechanical systems (MEMS) based inertial measurement units (IMUs), I^2^C multiplexer, and a 32-bit ARM Cortex-M4 microcontroller (Teensy^®^ 3.2, PJRC LLC, Sherwood, OR, USA). The PCB featured a designated space for a Teensy^®^ microcontroller (180 MHz, 256 KB RAM) and a port for a PCA9548 multiplexer operating on the I^2^C protocol. The Teensy^®^ microcontroller was selected owing to its superior clock frequencies and comprehensive integrated development environment (IDE) with advanced debugging capabilities, compared to analogous microcontrollers of comparable dimensions. Six IMUs (BNO055, Bosch Sensortec GmbH, Reutlingen, Germany), each incorporating a 3-axis gyroscope, 3-axis accelerometer, and 3-axis magnetometer, were interfaced with the multiplexer via connectors utilising I^2^C communication protocol. The system’s power management was facilitated through an onboard power connector, with operational power supplied by AA cells, providing the requisite voltage and current for sustained data acquisition.

IMU sensor drift presents a critical challenge in maintaining precision for biomedical motion tracking applications, with gyroscopic bias instability serving as the primary factor leading to cumulative errors in angular velocity measurements. A range of environmental conditions substantially influences the drift behaviour of these sensors. Our analysis demonstrated that electromagnetic interference (EMI) from surrounding electronic devices introduces considerable measurement uncertainties. Through strategic sensor positioning, we successfully minimised these EMI-induced errors in our system. Operating temperature fluctuations within the 12–20 °C range were found to influence both bias stability and sensor scale factor, which we effectively controlled through implemented compensation algorithms.

**Figure 1:**
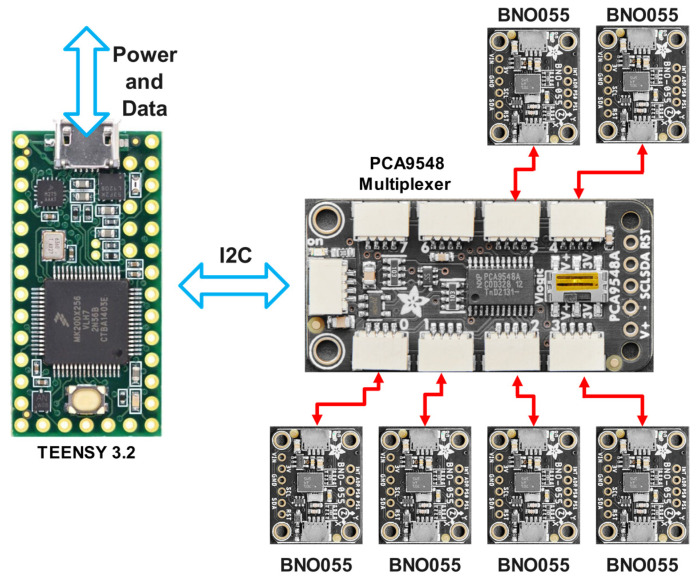
System architecture showing the data acquisition pathway.

In our system architecture, individual IMUs interface with the microcontroller via a multiplexer, enabling sequential data acquisition from each sensor at a sampling rate of 200 Hz (Figure 1). Raw measurements from each IMU are captured and stored independently in dedicated columns within a CSV file format. This approach ensures direct access to unprocessed sensor data, facilitating individual analysis of each IMU’s performance characteristics. The system’s data acquisition timing is governed by the multiplexer switching frequency, which has been optimised to maintain the 200 Hz sampling rate consistently across all sensors. We implemented a robust data collection protocol that maintains temporal synchronisation whilst preserving the independence of each sensor’s measurements.

**Figure 2:**
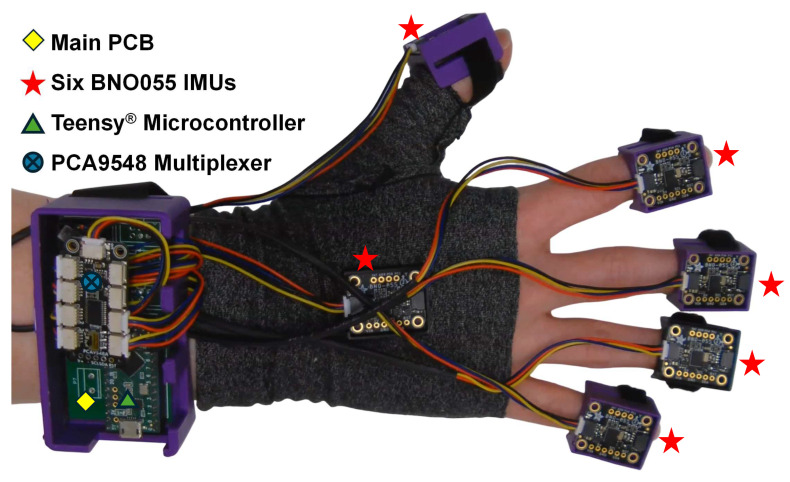
Overview of the glove system, showing key components: PCB, IMUs, microcontroller, and multiplexer.

As for the mechanical design, a spandex compression glove was modified by cutting along its width at the knuckles, to allow for customisability needed for accommodating the diverse range of hand differences (Figure 2). Five compartments to house IMUs for the five digits and a main compartment to house the remaining electronic componentry, including the PCB and the microcontroller, were manufactured. All these compartments were additively manufactured (fused deposition modelling) with PLA+ filament. Nylon webbing (25.4 mm wide) was used to secure the main compartment to the wrist, with both sides of the webbing threaded through designated areas of the compartment. During donning, the webbing was secured around the wrist with a hook-and-loop fastener patch (25.4 × 250.8 mm). The IMU compartments were secured to the dorsal side of the fingertips in a similar fashion, using hook-and-loop fasteners. A battery compartment holding two AA batteries was secured to the top of the main compartment using adhesive. An additional (sixth) IMU was secured to the back of the hand.

### 2.2. Software Design

**Figure 3:**
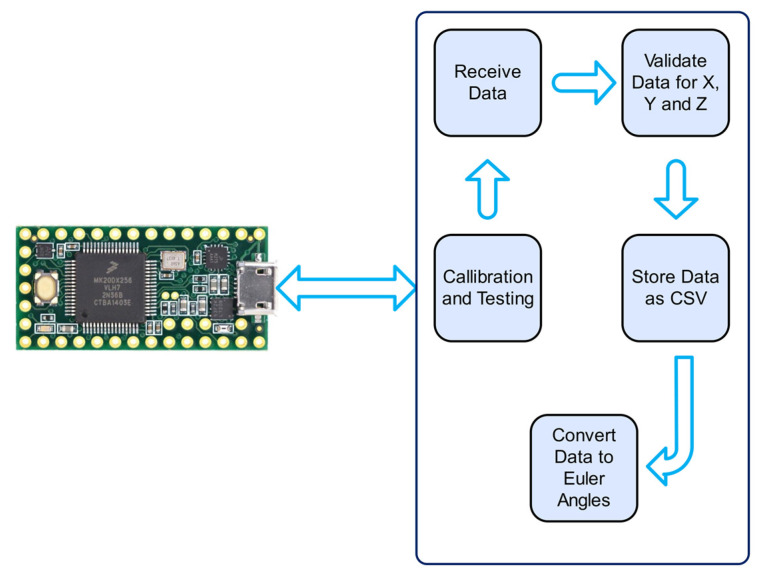
Software system architecture and data flow.

**Figure 4:**
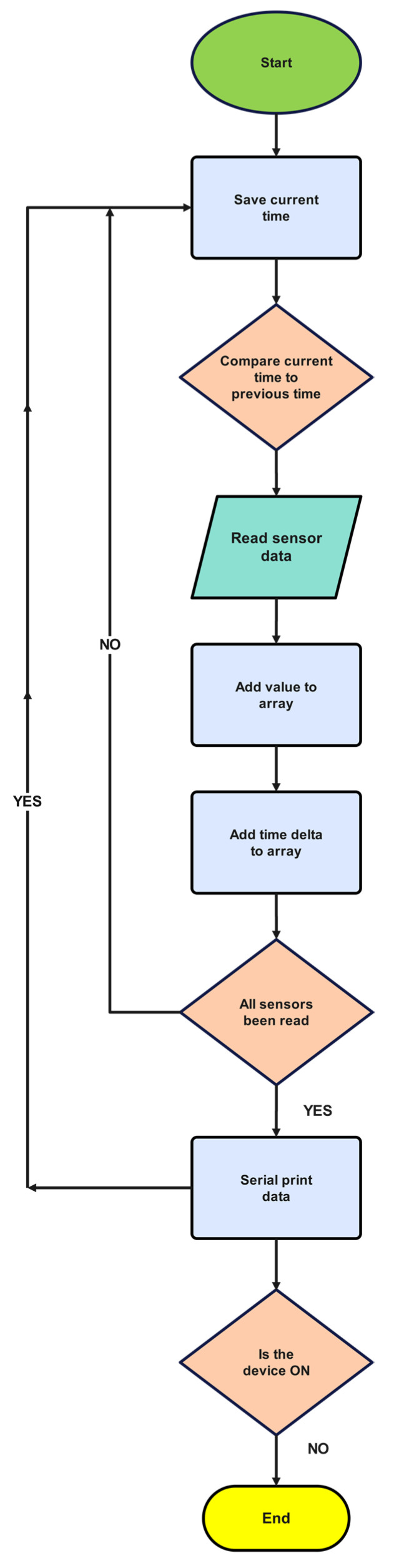
Flowchart showing the processes of the glove system’s firmware.

The embedded firmware was developed in C++ programming language to facilitate high-frequency data acquisition, concurrent multi-sensor sampling, and asynchronous serial data transmission requisite for Universal Serial Bus (USB) communication betwixt the data acquisition glove and a personal computer (Figure 3). Upon receipt of a data request command, the glove would transmit a structured data packet comprising three-dimensional Euler angles (pitch, roll, and yaw) and temporal measurements from all six MEMS-based inertial measurement units simultaneously at a sampling frequency of 200 Hz. Data transmission transpired when prompted by a connected PC via the virtual COM port operating at 115,200 baud rate, whereupon the streamed kinematic data from the glove would be stored in a comma-separated values (CSV) file, with each row containing a timestamp and 18 degrees of freedom (3 angles × 6 sensors) of motion data, facilitating subsequent visualisation of 3D hand movement (Figure 4).

### 2.3. Testing

To allow systematic evaluation of the IMU, it is important that the tests are both precise and reliable. While the evaluation of glove systems on human users is closer to real life, it has limitations such as inter-subject variability and difficulty in providing repeatable and precise movements, all of which prevent a more systematic evaluation. Instead, we adopted a robotic-arm-based testing approach which allowed us to perform precise, repeatable movements, without concerns for inter-subject variability and testing fatigue.

Hand differences are one of the most heterogenous medical conditions, manifesting in many forms [2]. A smart glove must be adaptable to a wide range of hand conditions and anatomies. Despite this, in its most fundamental form, the glove must be able to measure orientation between an IMU placed in the region of interest against a reference. Thus, in this study, we focused on evaluating the performance of an individual IMU sensor. To contextualise this, the rotations performed in these tests represented digit movements, particularly given the dominance of digits in hand movements, and the severe impact their impairment can have on overall hand function [1,2]. It is hoped that these robotic arm-based systematic tests on individual IMU sensors in isolation will truly discern their performance, allowing for more holistic evaluations of the glove system in the future.

**Figure 5:**
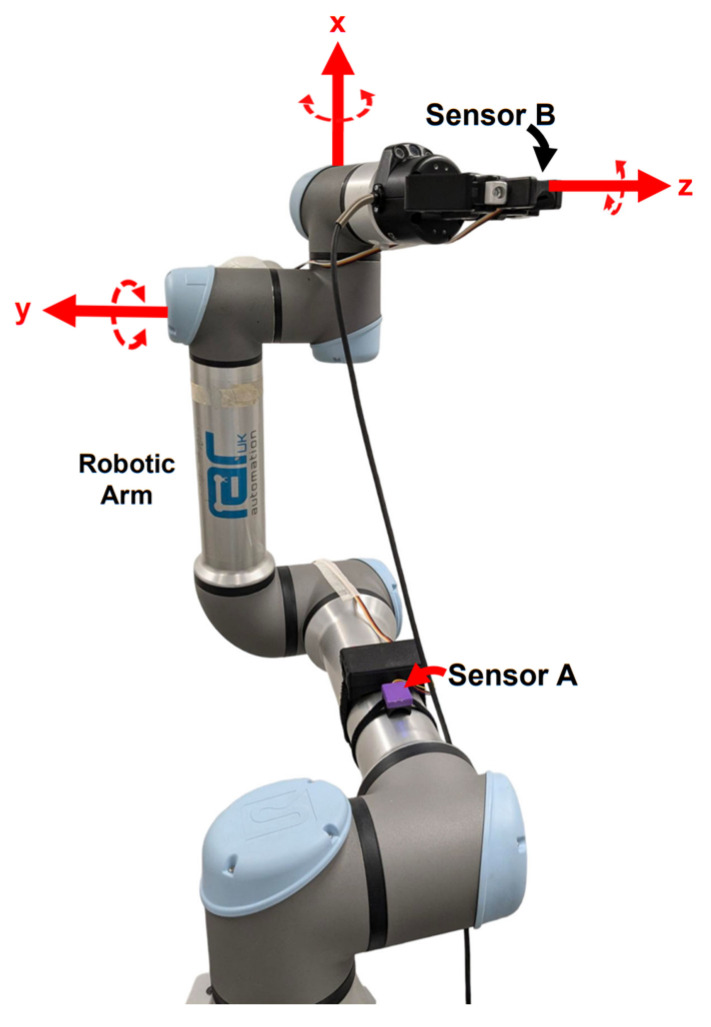
Experimental setup for validation of the IMU using the UR5e robotic arm, allowing rotation of sensor B in the x, y, and z axes shown.

To systematically evaluate the IMU sensor’s accuracy and reliability in measuring the three-dimensional range of motion of the digits, as compared to an industrial robotic arm, nine distinct tests were undertaken. Each trial commenced with mounting one of the glove’s IMU sensors (sensor A) onto a static surface of an industrial robotic arm (UR5e, Universal Robots, Novi, MI, USA). This sensor served as the reference point and represented a stationary wrist. A second IMU sensor was secured within the robotic arm’s gripper (2F-85, Robotiq, Lévis, QC, Canada), simulating the moving digit. Throughout all nine trials, it was this second, mobile IMU sensor (sensor B) that was manipulated by the robotic arm, whilst the first IMU sensor (sensor A) remained static (Figure 5).

**Figure 6:**
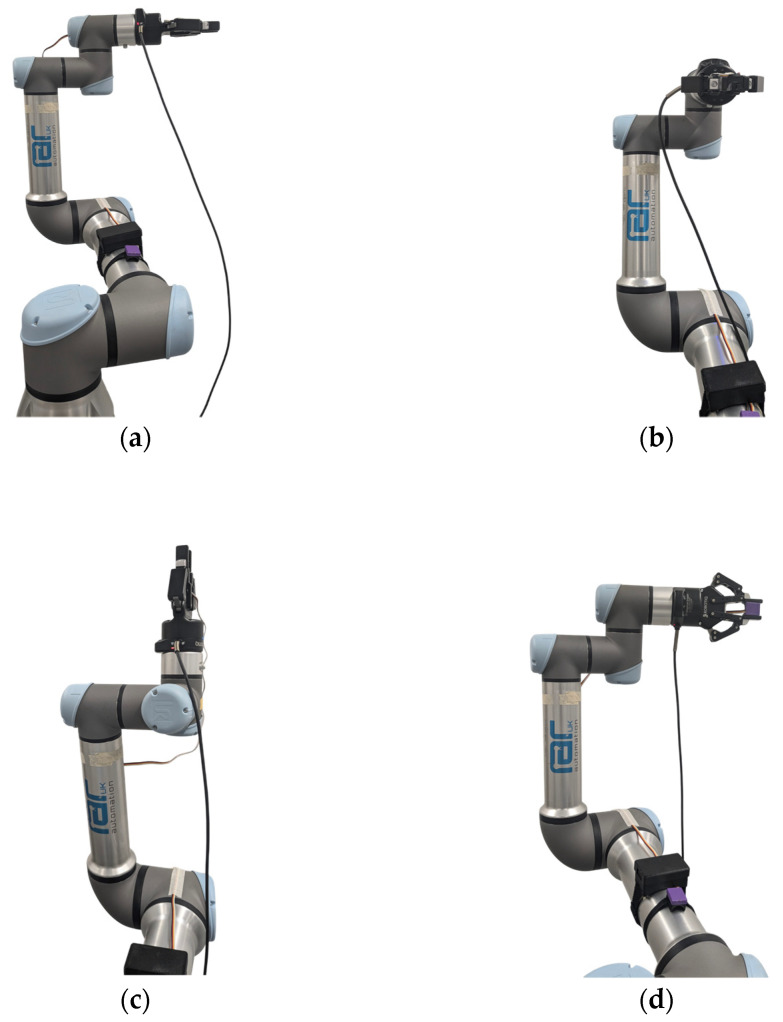
Robotic arm poses at rest (**a**) and at 90° rotation around *x*-axis (**b**), *y*-axis (**c**), and *z*-axis (**d**).

The nine tests involved three movements, which are as follows:

- Rotation of the sensor around the *x*-axis. This represented the medial/lateral abduction and adduction of the finger.
- Rotation of the sensor around the *y*-axis. This represented the extension, flexion, and hyperextension of the finger.
- Rotation of the sensor around the *z*-axis. This represented the supination and pronation of the finger.
- Each of these three movements was performed at three ranges: 90° to −90°, 10° to −10°, and 1° to –1°, resulting in a total of nine tests (Figure 6). Each test was performed twelve times to allow for statistical analysis.

Custom housings were designed and manufactured to host the two IMU sensors. The housing for the stationary sensor A allowed the sensor to be firmly rested on the robotic arm, with the use of hook-and-loop strips wrapped around the robotic arm. To further ensure that sensor A does not slide during tests, textured padding was placed in the housing for better grip. For sensor B, the housing allowed the gripper to hold the sensor perpendicular to the tool’s pinching direction and it prevented pressure from being imparted onto the sensor. This way, damage to sensor B was avoided.

The nine tests began with sensor B held at a neutral position (0°) by the robotic arm gripper. During the tests, the arm moved along programmed paths and stopped at set angles, using the RoboDK software (version 5.6.8, RoboDK Inc., Montreal, QC, Canada) via a local network connection. Targets were set using RoboDK and added to a program as pure joint rotations with no pathing active. The speed for the 90° to −90° and 10° to −10° tests was set to 10°/s tests, while for the 1° to −10° tests, it was set to 1°/s. For all nine tests, sensor B rotated in the following order: neutral position, positive extreme, negative extreme, and neutral position. For example, for the test involving 90 to −90° rotation around *x*-axis, the order of rotations was as follows: 0° (neutral position), 90° (positive extreme), −90° (negative extreme), and 0° (neutral position).

A Python script was developed to record the motion of a robot arm. The program used the difference in joint angles recorded from the robot arm to schedule requests for data from the glove. When a significant joint movement was made (>0.01° at any joint), a request for data from the glove was made and recorded in a .csv file along with the robot joint positions. All joint data were recorded for the robot arm and the two IMU sensors.

For trials of all nine tests, Kruskal–Wallis test was performed to determine if there was a statistically significant difference between the robot arm and the IMU sensors. The null hypothesis (H_0_) for this test was that there was no statistically significant difference between the robot arm and the IMU sensors. Then, for each trial, Root Mean Square Error (RMSE) and Mean Absolute Error (MAE) were determined. For each test, correlation heatmap, and agreement matrix were created to help determine the pair-wise and inter-trial correlations. All statistical analyses were performed using Python.

## 3. Results

**Figure 7:**
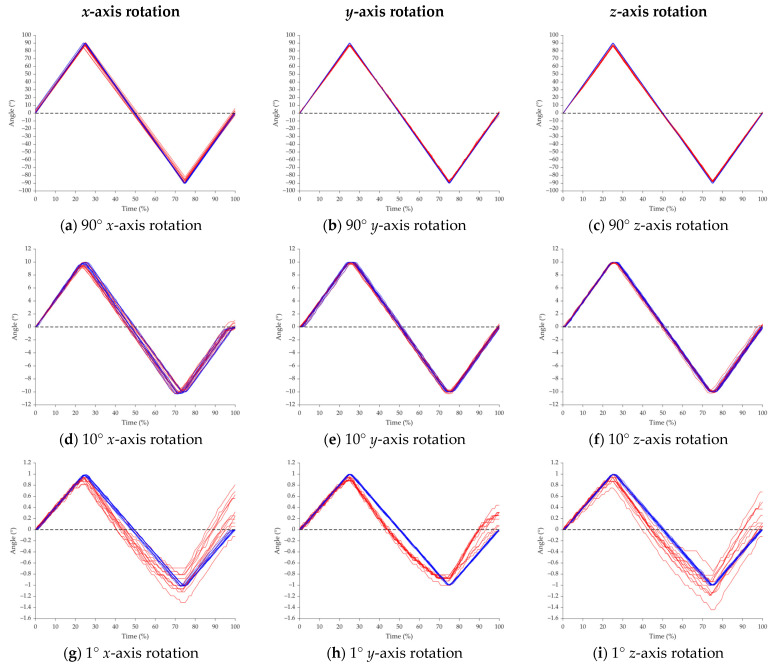
Angle vs. Time plots measured from the IMU sensors (red) and robotic arms (blue), for all twelve trials of the 90° (**a**–**c**), 10° (**d**–**f**), and 1° (**g**–**i**) *x*-axis rotation, *y*-axis rotation, and *z*-axis rotation tests.

Overall, the IMU sensors showed strong concordance with the robotic arm (Figure 7), suggesting robust measurement validity. The Kruskal–Wallis analyses yielded *p*-values exceeding 0.95 throughout all trials of the nine tests, lending support to the null hypothesis and demonstrating that the measurements from the IMU sensors and robotic arm were statistically equivalent (Table 1). This high degree of statistical similarity indicates minimal systematic bias between the two measurement systems. The agreement between RMSE (Root Mean Square Error) and MAE (Mean Absolute Error) values suggests that the measurement system exhibits consistent performance across the full range of motion, with negligible outliers or systematic drift that might otherwise cause discrepancies between these error metrics (Table 2). This stability across the motion range implies that the IMU sensors maintain their accuracy regardless of the position or velocity of movement being measured.

**Figure 8:**
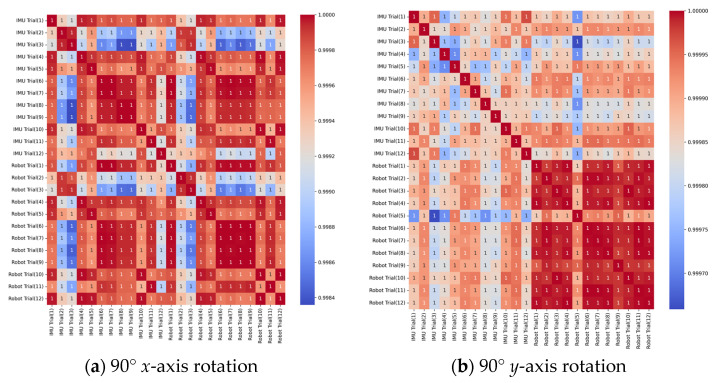
Correlation heatmaps for all twelve trials of the (**a**) 90° *x*-axis and (**b**) 90° *y*-axis rotation tests.

The correlation heatmaps revealed that correlation values exceeded 0.91 across all trial pairs of the ten tests, indicating strong positive correlations between the robot and IMU sensors (Figure 8; see Supplementary Figures S1–S7 for correlation heatmaps for the remaining tests). This high correlation coefficient suggests good measurement consistency between the two systems. Moreover, the inter-trial correlations demonstrated equally robust positive relationships, also exceeding 0.91, which indicates high test-retest reliability. These findings were corroborated by the agreement matrices (Supplementary Figures S8–S16), which demonstrated high agreement (<1°) for 24 trials and moderate agreement (1–10°) for 12 trials across all tests. A small subset of four trials exhibited low agreement (>10°), suggesting isolated instances where measurement discrepancies occurred. The predominance of high and moderate agreement provides compelling evidence for the overall measurement precision and reliability of the IMU sensor system relative to the robotic reference standard.

In terms of practical implications in a clinical environment, these statistical analysis findings imply that IMU sensors can allow the capture of fine digit movements in abduction, adduction, extension, flexion, supination, and pronation. Thus, the IMU-based glove can be integrated into the existing clinical hand assessments such as the Jebson–Taylor Hand Function Test which rely heavily on tasks based on digit movements [11]. Further, given the high agreement (<1°) between the IMU sensor and robotic arm, the IMU-based glove could potentially allow clinicians to discern and diagnose a wide range of hand differences.

## 4. Discussion

In recent years, there has been increased interest in monitoring 3D hand movement. Previous studies have focused on the potential application of smart gloves for assessing hand function but often with a focus on specific conditions such as stroke [22,23,24]. Information regarding the general accuracy and reliability of these gloves in measuring joint rotation is missing in the literature [17]. By systematically evaluating the glove sensor’s accuracy and reliability in measuring the 3D finger joint angles against an industrial robotic arm, we obtained a better understanding of the glove’s ability to measure rotation in three axes (x, y, and z), simulating finger extension-flexion, supination-pronation, and abduction-adduction. This contributes to the current knowledge by demonstrating their effectiveness for potential applications in assessing hand differences.

The glove sensor was able to record 90°, 10°, and 1° rotation in all three directions. Extension-flexion, supination-pronation, and abduction-adduction of digits are fundamental movements of the human hand, and their combinations enable humans to perform complex tasks. The glove sensor’s performance in the three directions was similar, demonstrating that it can be used not only for measuring the finger’s extension-flexion ROM, which is a common clinical measurement. It can also be used to measure the finger’s deviations in abduction-adduction and supination-pronation. These findings, along with the fact that relatively large angle ranges (180°) in all three directions were evaluated in this study, indicate that the glove sensor is versatile in its application for assessing hands with large differences from the anatomical hand. Hand differences can both increase and decrease the ROMs of the fingers as well as alter the resting position of the hand [2]. Thus, these sensors allow clinicians to capture these movements in the clinic and provide more quantitative data to supplement the current qualitative assessments and hand function tests such as the Jebsen–Taylor Hand Function Test [11]. This additional information will allow clinicians to make more informed clinical decisions and objectively evaluate the effectiveness of interventions. Further, the deployment of the glove outside the clinical setting would also enable remote monitoring of hand function. Such remote monitoring would provide a more accurate representation of the patient’s hand function in real life. Currently, there is no standard method for evaluating glove sensors for hand differences, with the common approach being testing on human subjects [22,23,24]. In the current study, the robotic arm provided a repeatable method for testing the sensor on multiple axes in isolation. It allowed us to eliminate factors such as intra- and inter-subject variability, subject biases, and subject fatigue, that are associated with human testing. Thus, we recommend the use of a robotic arm for future studies of smart gloves.

As for the limitations of the current study, first, while the use of a robotic arm provided a highly repeatable setup for evaluating the sensor, the glove system was not tested on human subjects. Second, the study focused on testing the sensor’s performance for isolated finger rotations but did not assess simultaneous movements of all five digits. The ability to capture complex, multi-digit, multi-axial hand movements remains unexplored. Third, the glove sensor was not evaluated on unhealthy subjects, such as individuals with neuromuscular disorders or hand injuries, which limits the generalisability of the findings to clinical applications. Lastly, the emphasis of this work was on real-time sensor data and repeatability, potentially overlooking broader aspects of usability or long-term durability under diverse conditions.

To address the aforementioned limitations, future research studies should incorporate testing on human subjects. This will enable the evaluation of the glove sensor in realistic conditions, accounting for inter-subject variability and the complexity of natural hand movements. Additionally, testing all five digits simultaneously could provide a comprehensive assessment of the glove’s ability to capture intricate hand functions during dynamic, multi-finger tasks. Such experiments should include a broader participant pool, encompassing both healthy and unhealthy subjects, to explore the glove’s applicability for diagnostic or therapeutic purposes. Finally, integrating assessments of usability, durability, and long-term performance could strengthen the practical value of the glove sensor for both research and clinical contexts. In the clinical context, the glove can potentially be used to monitor hand function in patients with neuromuscular disorders, track recovery progress after hand surgeries, or evaluate motor impairments in conditions such as arthritis or stroke. The glove system’s integration into rehabilitation programs could provide real-time feedback to patients and therapists, enabling more personalised and effective therapy interventions.

## 5. Conclusions

In conclusion, this study has demonstrated the accuracy and reliability of IMU sensors in measuring three-dimensional joint rotations, with the results aligning closely with an industrial robotic arm across various test movements. These findings indicate good potential application of IMUs in smart gloves for tracking key digit movements during extension-flexion, supination-pronation, and abduction-adduction. Such IMU-based smart gloves could potentially offer clinicians more accurate and reliable digit motion data to supplement the current clinical assessment for hand differences.

## Figures and Tables

**Table 1:** *p*-values of all trials of the *x*-, *y*-, and *z*-axis rotation tests, as determined by the Kruskal–Wallis test.

Trial Number	*x*-Axis Rotation			*y*-Axis Rotation			*z*-Axis Rotation		
	90°	10°	1°	90°	10°	1°	90°	10°	1°
1	0.939	0.880	0.406	0.880	0.959	0.959	0.979	0.899	0.632
2	0.762	0.840	0.979	0.979	0.959	0.939	0.880	0.668	0.743
3	0.841	0.939	0.546	0.979	0.979	0.919	0.979	0.762	0.537
4	0.772	0.820	0.743	0.939	0.705	0.860	0.979	0.880	0.571
5	0.772	0.919	0.724	0.899	0.801	0.979	0.979	0.939	0.496
6	1.000	0.850	0.504	0.959	0.801	0.880	0.899	0.880	0.899
7	0.909	0.899	0.562	0.860	0.801	0.959	0.880	0.949	0.899
8	0.734	0.969	0.959	0.919	0.939	0.650	0.919	0.959	0.144
9	0.588	0.830	0.724	0.860	1.000	0.762	0.939	0.959	0.413
10	0.959	0.979	0.339	0.880	0.840	0.705	0.919	1.000	0.899
11	0.909	0.959	0.743	0.959	0.929	0.969	0.979	0.959	0.979
12	0.919	0.840	0.632	0.840	0.820	0.801	0.959	0.919	0.860

**Table 2:** Root Mean Square Error (RMSE) and Mean Absolute Error (MAE), in parentheses, for all trials of the *x*-, *y*-, and *z*-axis rotation tests.

Trial Number	*x*-Axis Rotation			*y*-Axis Rotation			*z*-Axis Rotation		
	90°	10°	1°	90°	10°	1°	90°	10°	1°
1	1.862 (1.570)	0.358 (0.286)	0.341 (0.221)	1.993 (1.791)	0.198 (0.158)	0.200 (0.161)	1.992 (1.839)	0.132 (0.106)	0.269 (0.187)
2	3.548 (2.905)	0.367 (0.275)	0.239 (0.184)	1.388 (0.967)	0.182 (0.156)	0.216 (0.175)	2.407 (2.071)	0.249 (0.213)	0.171 (0.141)
3	3.126 (2.470)	0.395 (0.345)	0.251 (0.151)	2.203 (1.983)	0.116 (0.091)	0.213 (0.170)	2.913 (2.603)	0.154 (0.118)	0.151 (0.116)
4	2.622 (2.147)	0.538 (0.386)	0.253 (0.191)	1.486 (1.049)	0.336 (0.301)	0.179 (0.144)	2.696 (2.370)	0.154 (0.113)	0.157 (0.118)
5	2.904 (2.373)	0.420 (0.339)	0.140 (0.109)	1.603 (1.322)	0.213 (0.164)	0.202 (0.163)	2.592 (2.270)	0.219 (0.190)	0.185 (0.143)
6	1.918 (1.635)	0.261 (0.189)	0.288 (0.204)	1.357 (1.021)	0.178 (0.139)	0.130 (0.107)	2.608 (2.278)	0.206 (0.165)	0.222 (0.174)
7	2.048 (1.736)	0.295 (0.215)	0.244 (0.199)	1.767 (1.452)	0.178 (0.139)	0.213 (0.177)	2.220 (1.965)	0.142 (0.113)	0.219 (0.16)
8	3.337 (2.647)	0.245 (0.207)	0.129 (0.107)	1.444 (1.080)	0.094 (0.073)	0.152 (0.131)	2.148 (1.807)	0.157 (0.124)	0.360 (0.309)
9	5.262 (4.679)	0.348 (0.285)	0.141 (0.110)	1.419 (1.146)	0.165 (0.146)	0.130 (0.102)	1.920 (1.609)	0.158 (0.122)	0.190 (0.150)
10	1.973 (1.672)	0.180 (0.151)	0.243 (0.203)	1.592 (1.411)	0.155 (0.118)	0.214 (0.175)	1.838 (1.591)	0.114 (0.094)	0.136 (0.108)
11	1.983 (1.698)	0.251 (0.187)	0.172 (0.141)	1.457 (1.209)	0.184 (0.139)	0.230 (0.183)	1.900 (1.739)	0.163 (0.138)	0.209 (0.163)
12	2.120 (1.705)	0.295 (0.203)	0.151 (0.119)	2.238 (2.015)	0.242 (0.201)	0.222 (0.179)	1.850 (1.654)	0.253 (0.194)	0.155 (0.135)

## Data Availability

The original contributions presented in this study are included in the article/Supplementary Materials. Further inquiries can be directed to the corresponding author.

## Supplementary Materials

The following supporting information can be downloaded at https://www.mdpi.com/article/10.3390/s25010002/s1, Figure S1: Correlation heatmaps for all twelve trials of the 90° *z*-axis rotation test; Figure S2: Correlation heatmaps for all twelve trials of the 10° *x*-axis rotation test; Figure S3: Correlation heatmaps for all twelve trials of the 10° *y*-axis rotation test; Figure S4: Correlation heatmaps for all twelve trials of the 10° *z*-axis rotation test; Figure S5: Correlation heatmaps for all twelve trials of the 1° *x*-axis rotation test; Figure S6: Correlation heatmaps for all twelve trials of the 1° *y*-axis rotation test; Figure S7: Correlation heatmaps for all twelve trials of the 1° *z*-axis rotation test; Figure S8: Agreement matrix for all twelve trials of the 90° *x*-axis rotation test; Figure S9: Agreement matrix for all twelve trials of the 90° *y*-axis rotation test; Figure S10: Agreement matrix for all twelve trials of the 90° *z*-axis rotation test; Figure S11: Agreement matrix for all twelve trials of the 10° *x*-axis rotation test; Figure S12: Agreement matrix for all twelve trials of the 10° *y*-axis rotation test; Figure S13: Agreement matrix for all twelve trials of the 10° *z*-axis rotation test; Figure S14: Agreement matrix for all twelve trials of the 1° *x*-axis rotation test; Figure S15: Agreement matrix for all twelve trials of the 1° *y*-axis rotation test; Figure S16: Agreement matrix for all twelve trials of the 1° *z*-axis rotation test.
