# Supplementary figures and images for: Systematic Evaluation of IMU Sensors for Application in Smart Glove System for Remote Monitoring of Hand Differences
Source: Sensors (Basel). 2024 Dec 24;25(1):2. doi: 10.3390/s25010002 (PMC11722738; doi:10.3390/s25010002)

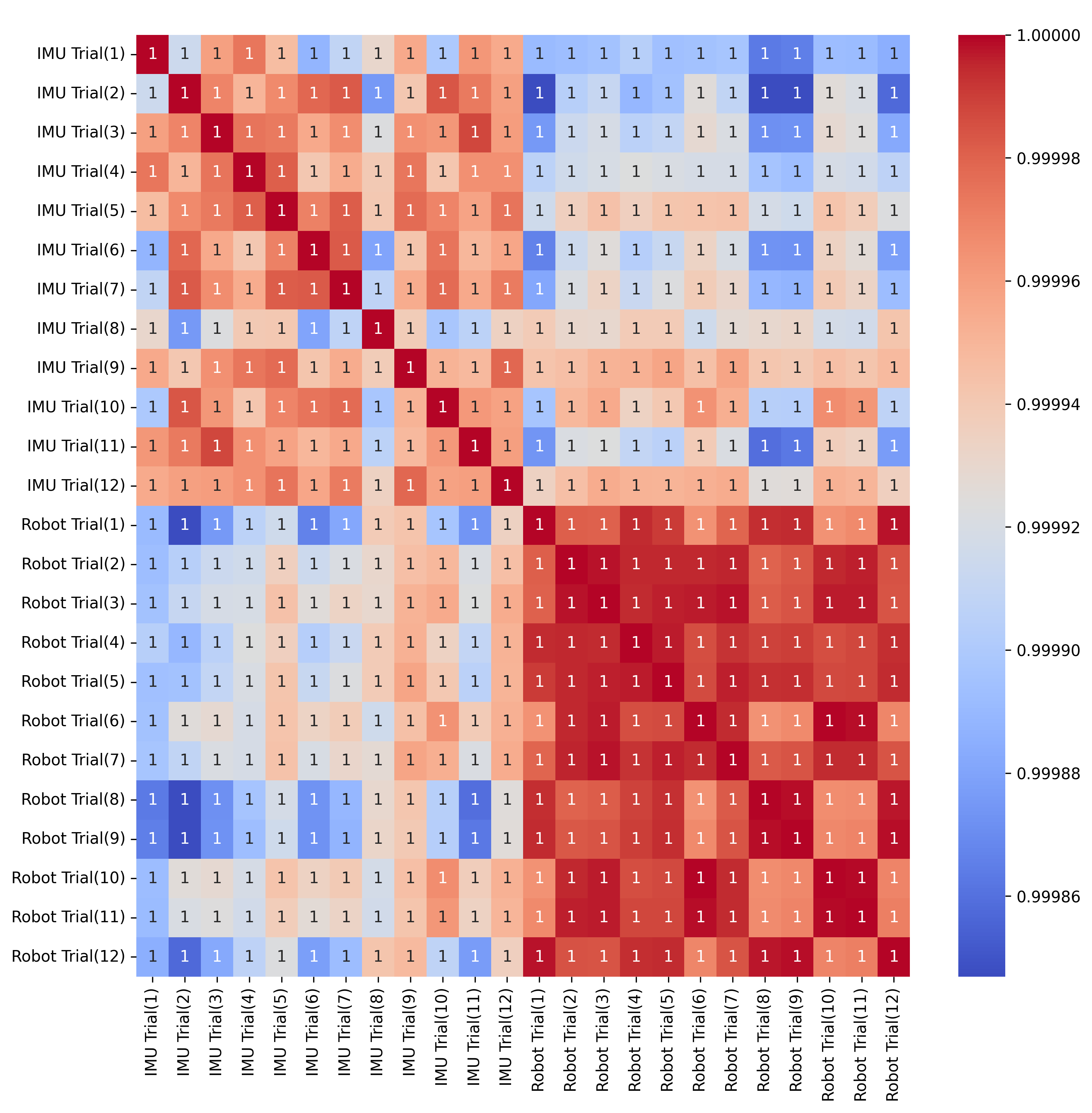

Supplement: Supplementary file 1 [file sensors-25-00002-s001.zip › Supplementary Materials/S1.png]

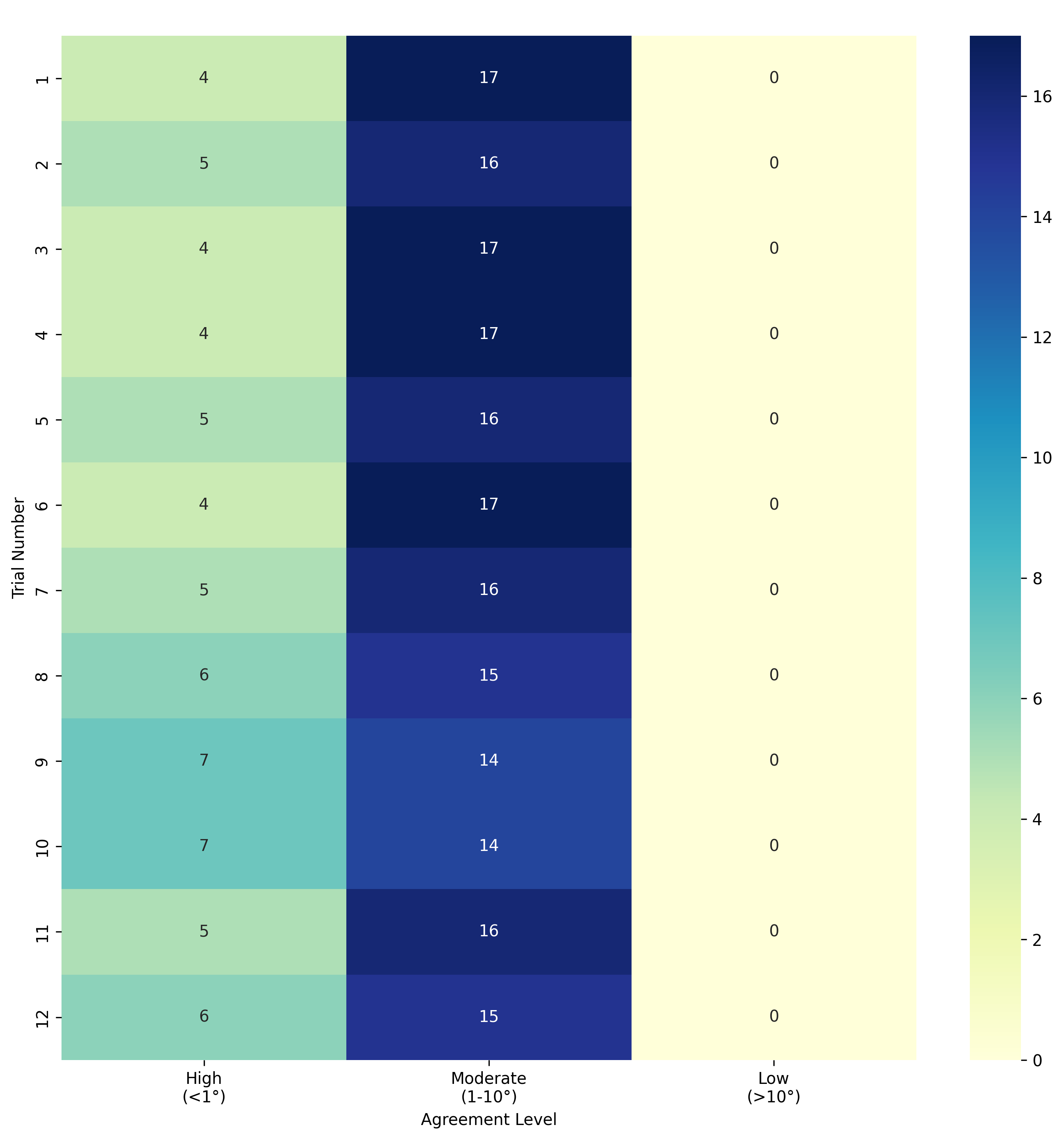

Supplement: Supplementary file 1 [file sensors-25-00002-s001.zip › Supplementary Materials/S10.png]

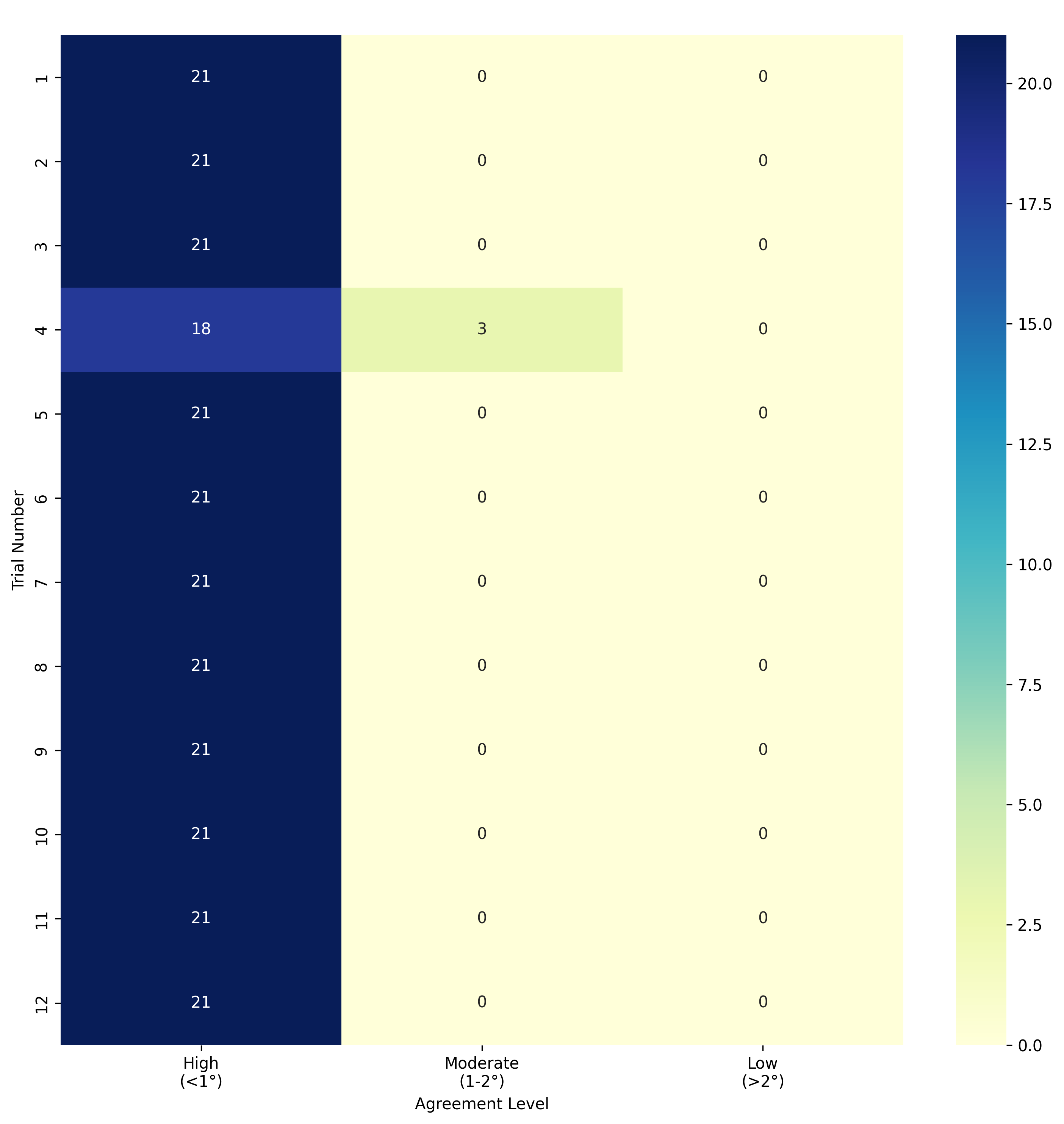

Supplement: Supplementary file 1 [file sensors-25-00002-s001.zip › Supplementary Materials/S11.png]

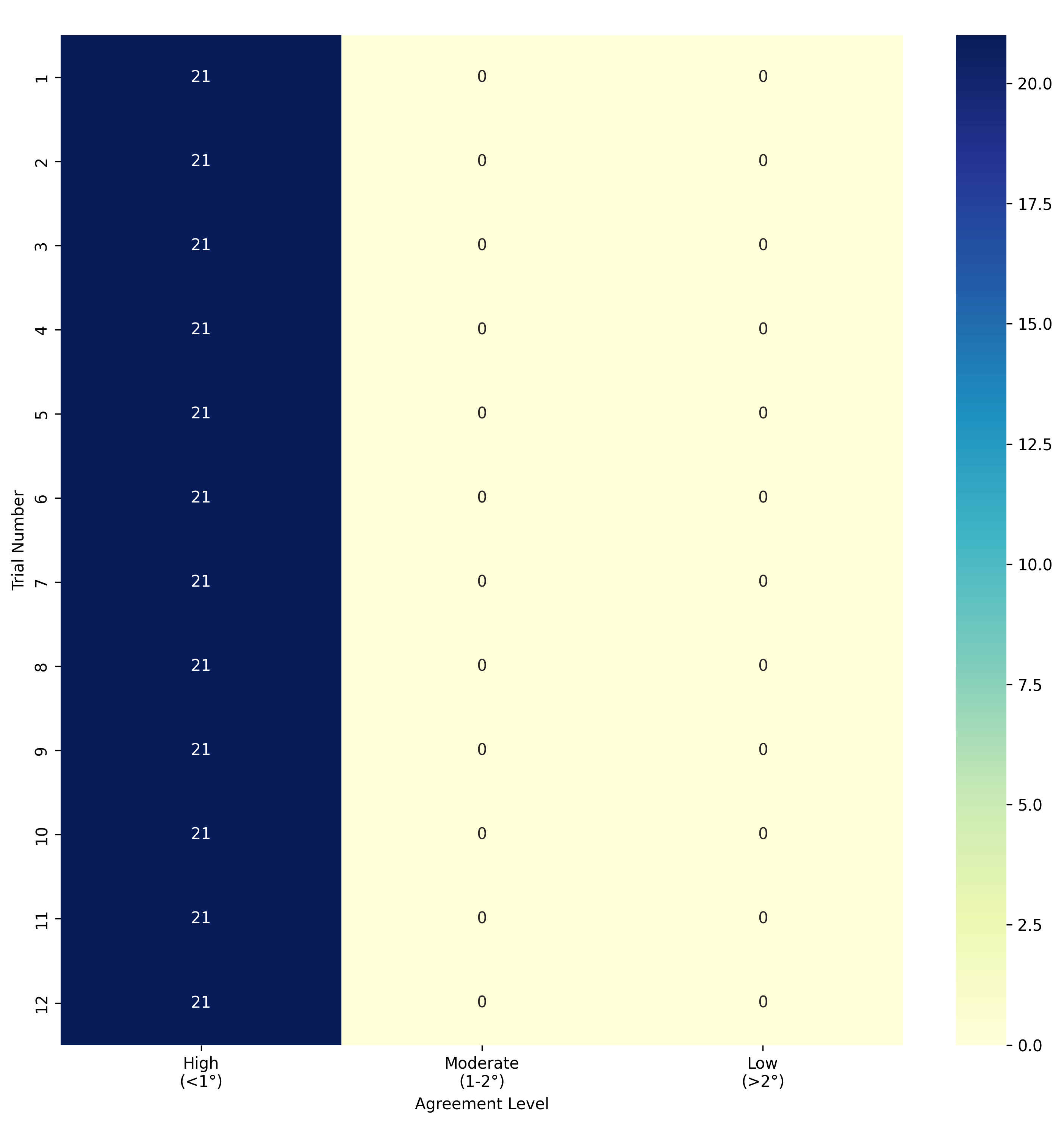

Supplement: Supplementary file 1 [file sensors-25-00002-s001.zip › Supplementary Materials/S12.png]

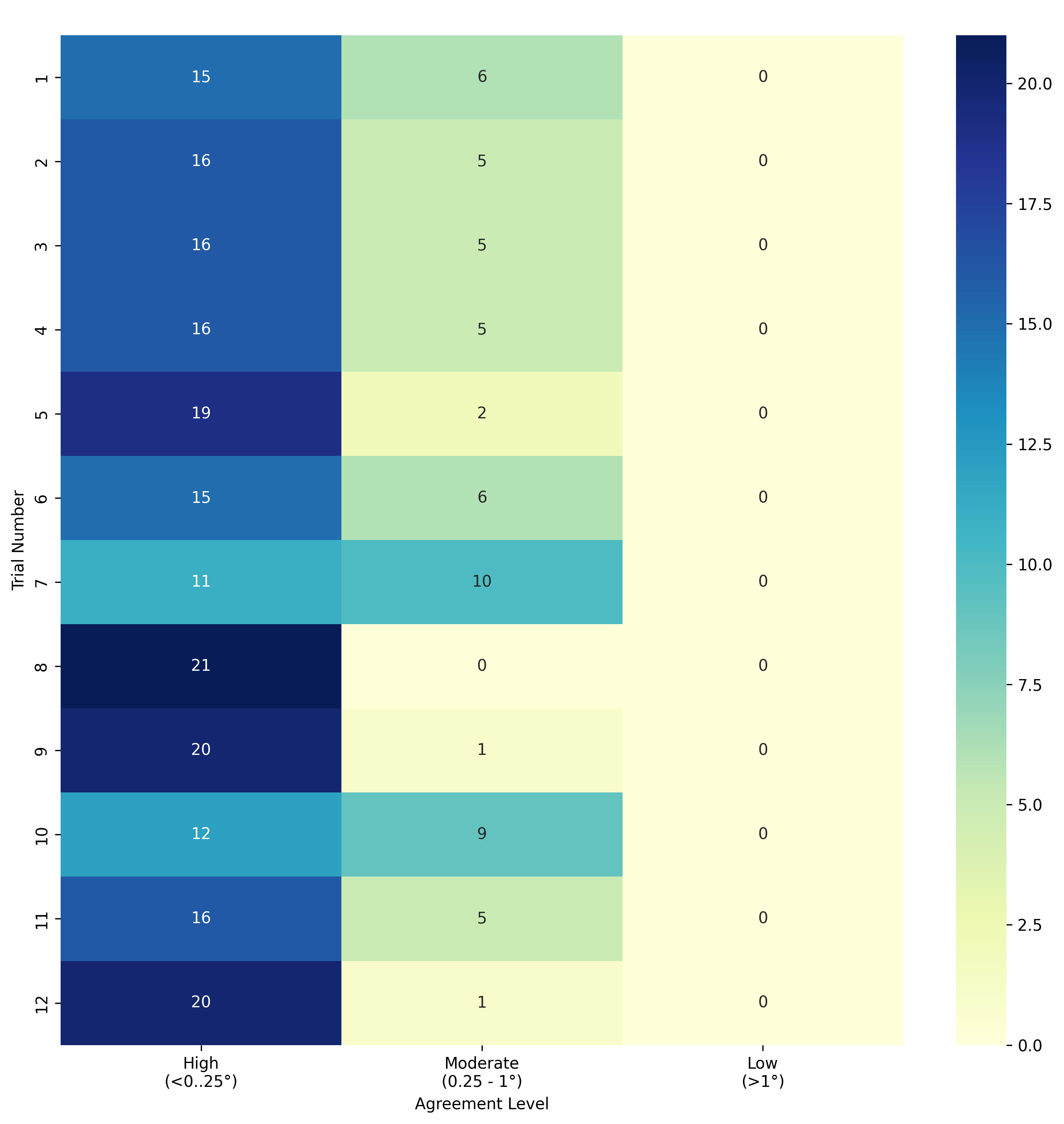

Supplement: Supplementary file 1 [file sensors-25-00002-s001.zip › Supplementary Materials/S14.png]

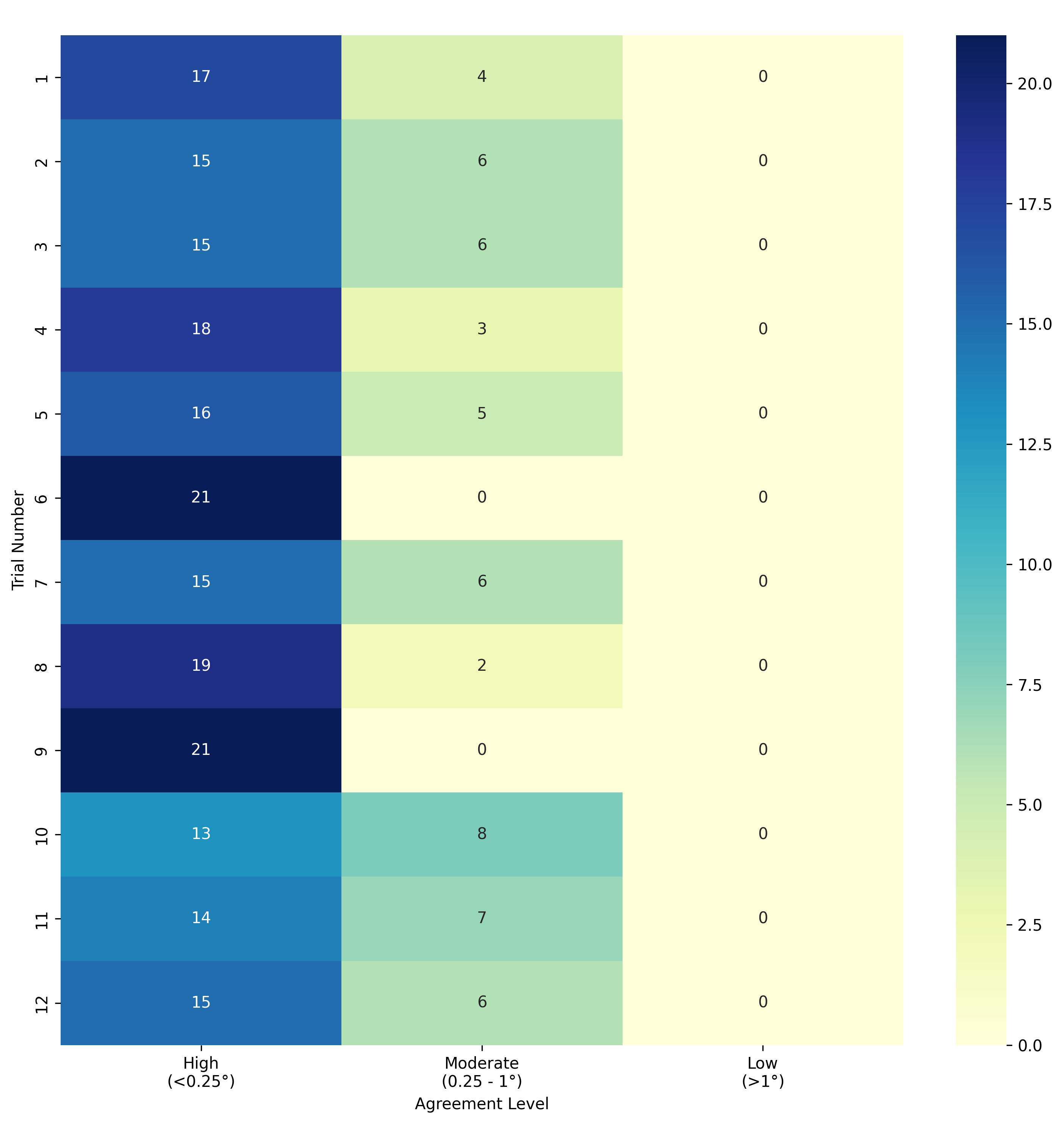

Supplement: Supplementary file 1 [file sensors-25-00002-s001.zip › Supplementary Materials/S15.png]

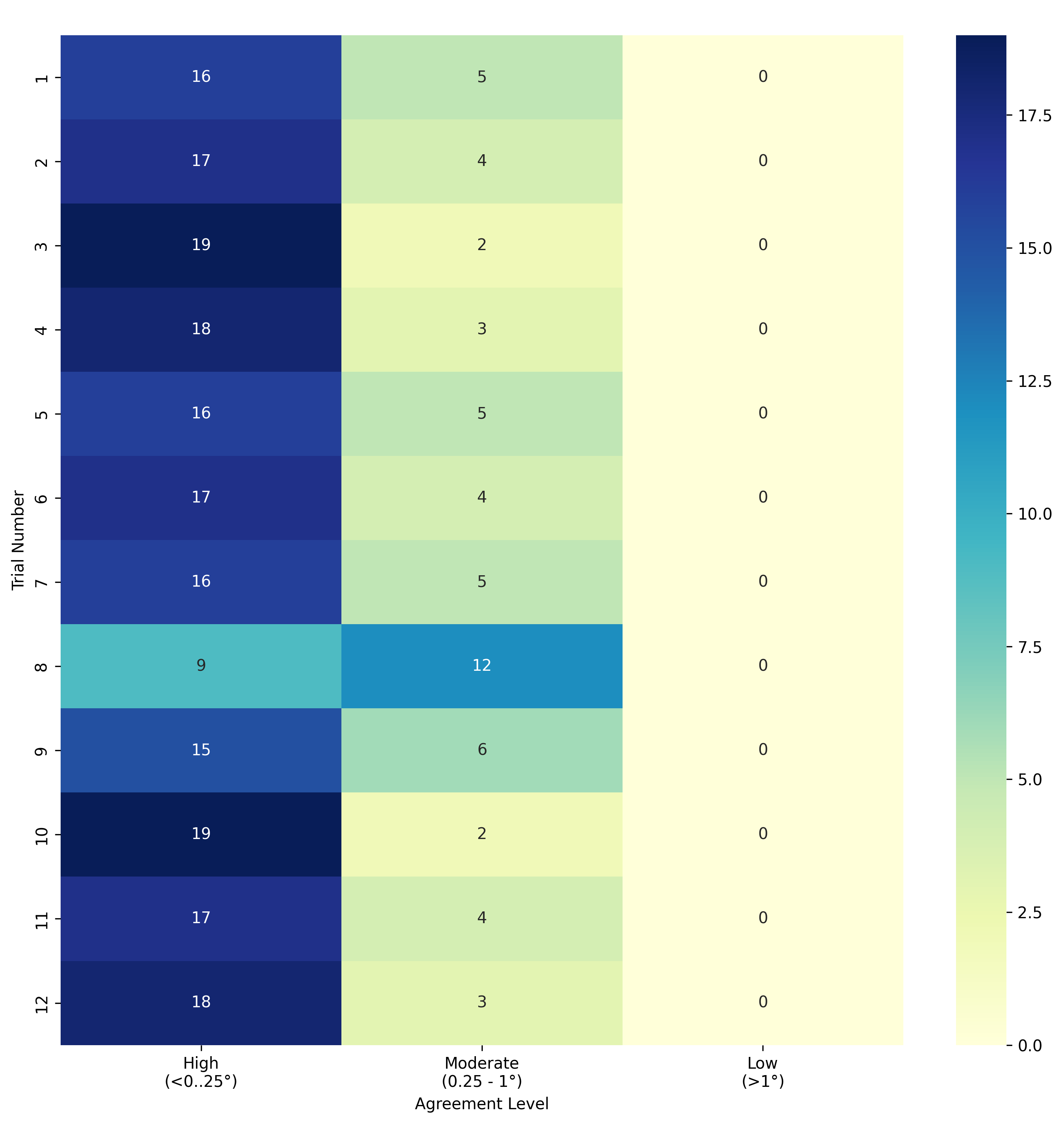

Supplement: Supplementary file 1 [file sensors-25-00002-s001.zip › Supplementary Materials/S16.png]

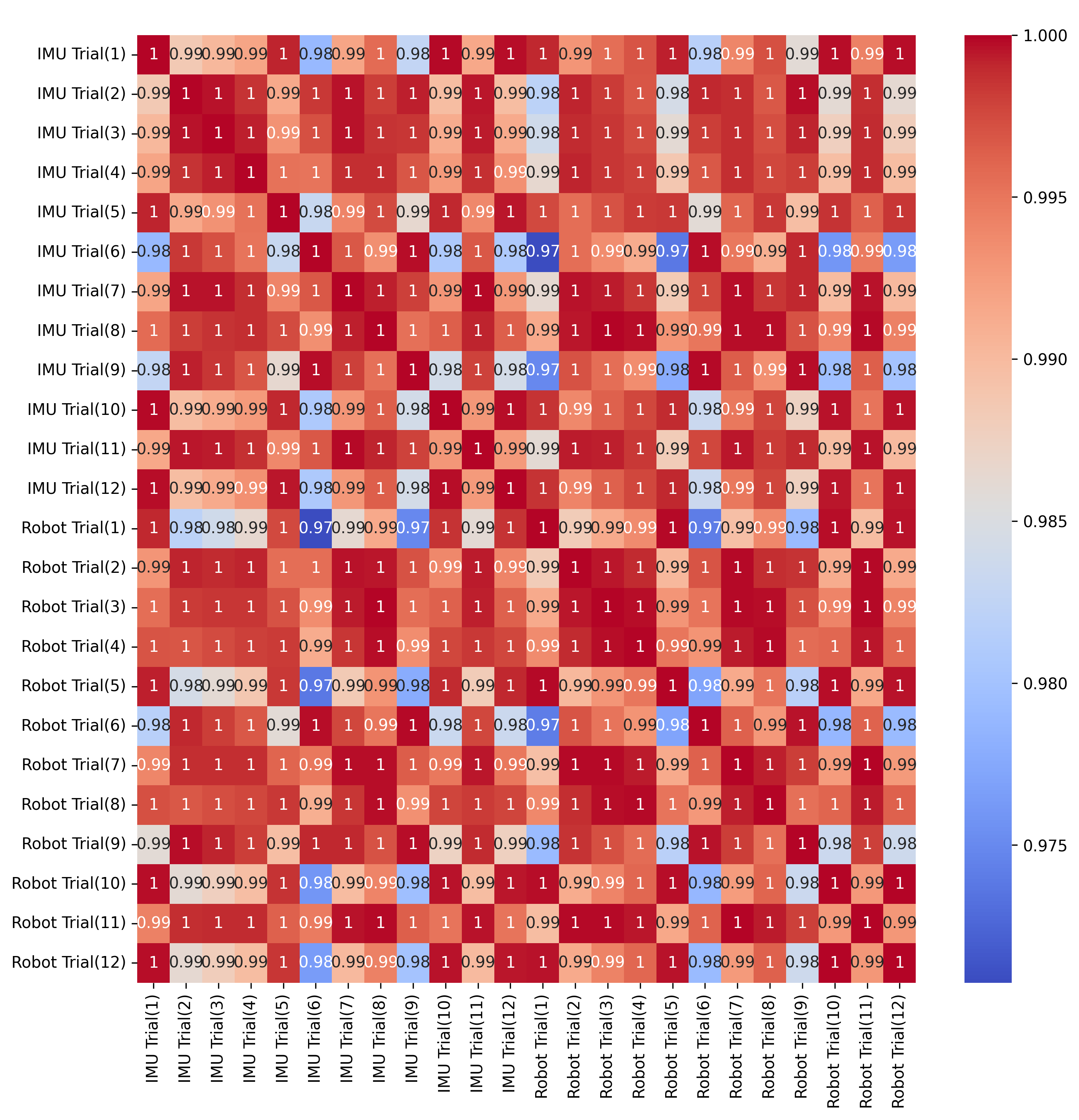

Supplement: Supplementary file 1 [file sensors-25-00002-s001.zip › Supplementary Materials/S2.png]

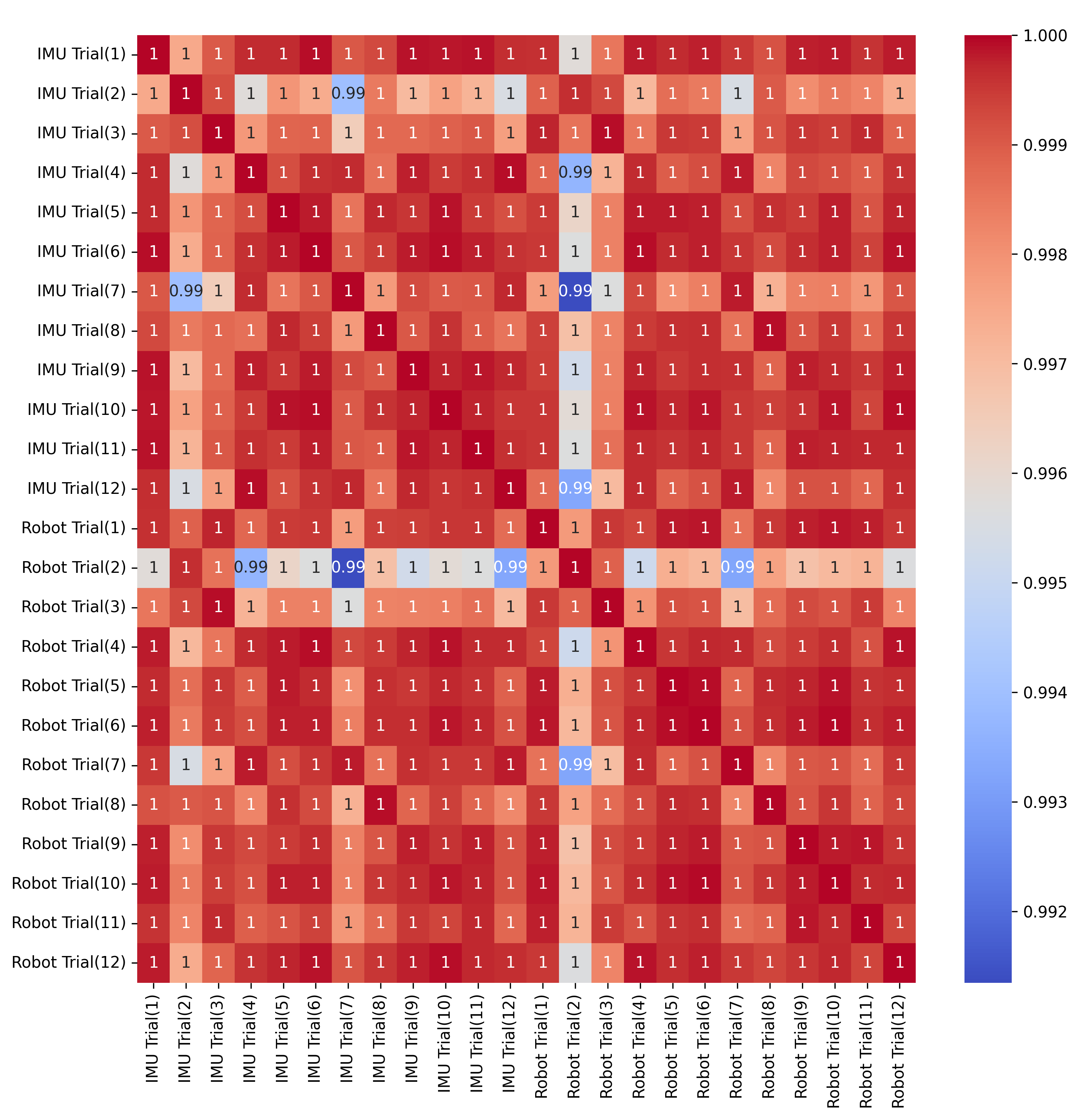

Supplement: Supplementary file 1 [file sensors-25-00002-s001.zip › Supplementary Materials/S3.png]

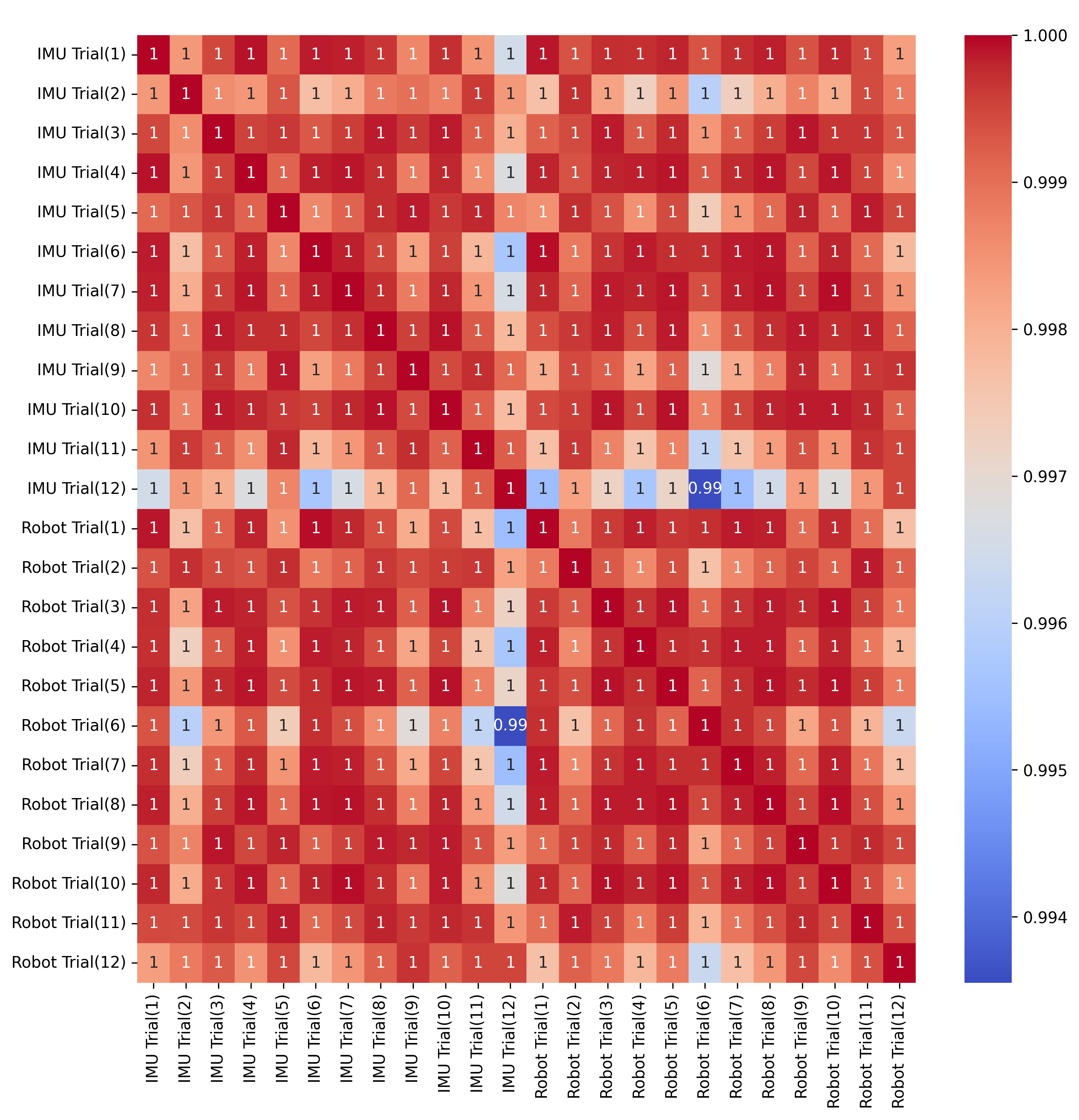

Supplement: Supplementary file 1 [file sensors-25-00002-s001.zip › Supplementary Materials/S4.png]

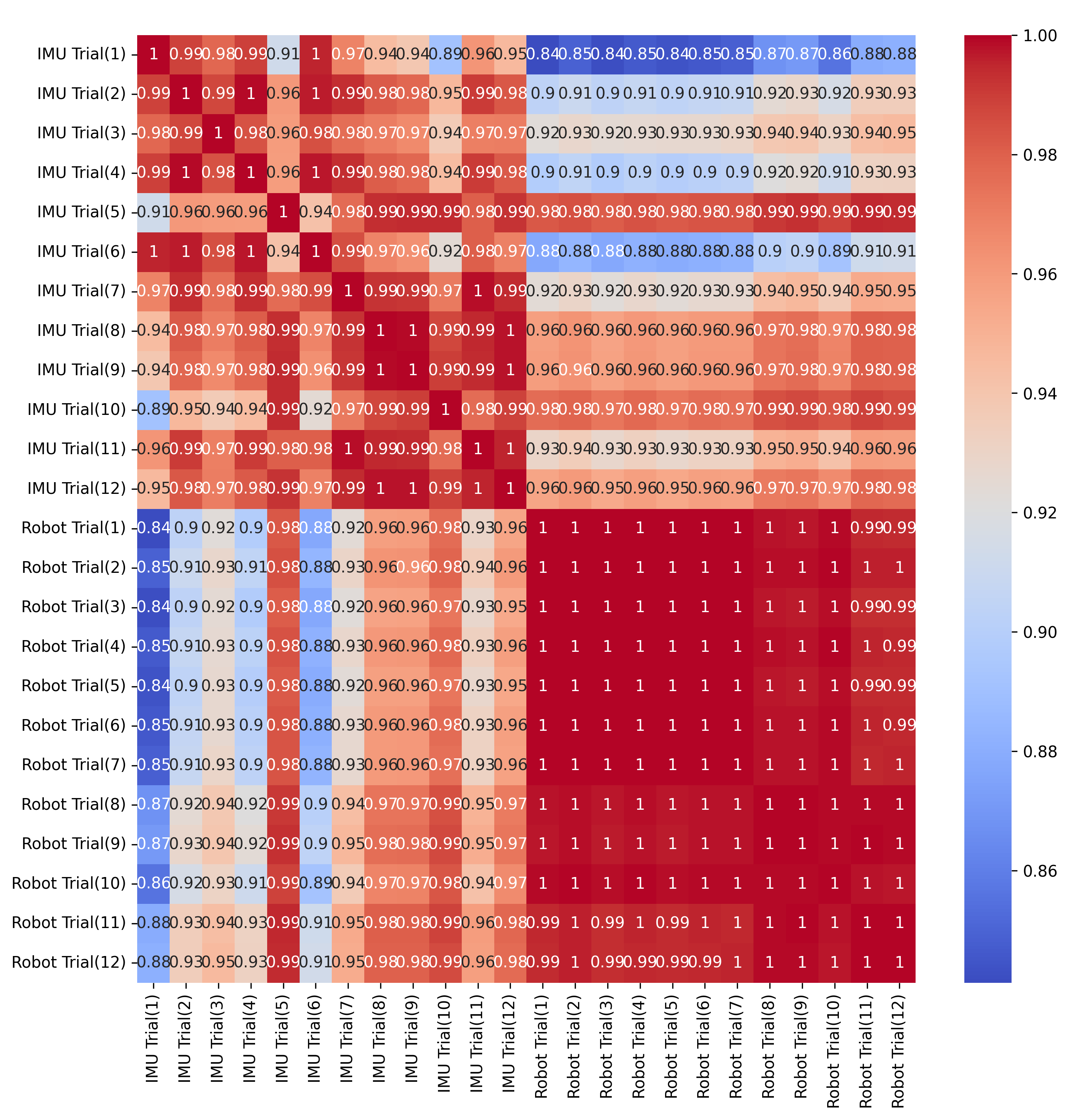

Supplement: Supplementary file 1 [file sensors-25-00002-s001.zip › Supplementary Materials/S5.png]

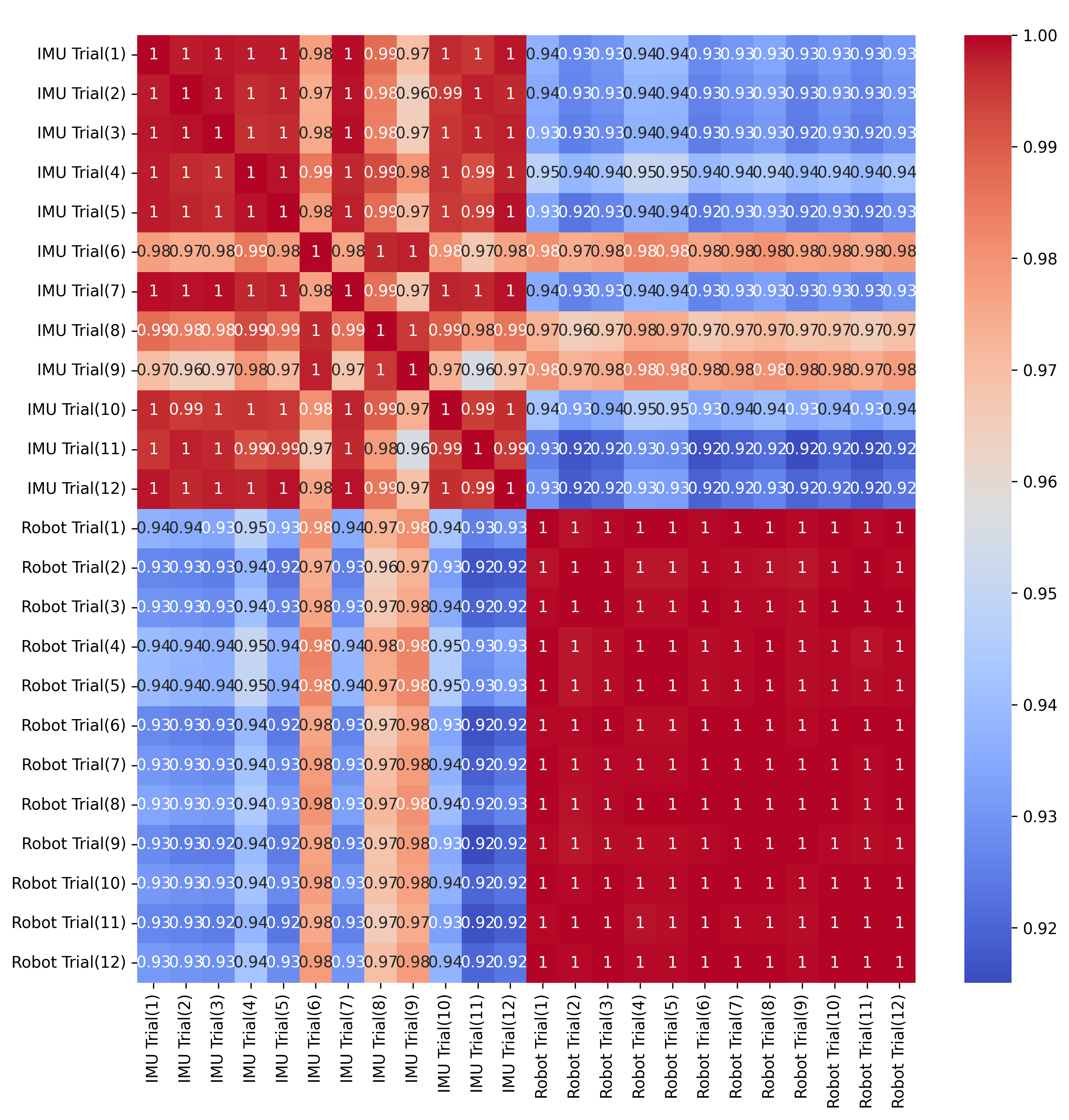

Supplement: Supplementary file 1 [file sensors-25-00002-s001.zip › Supplementary Materials/S6.png]

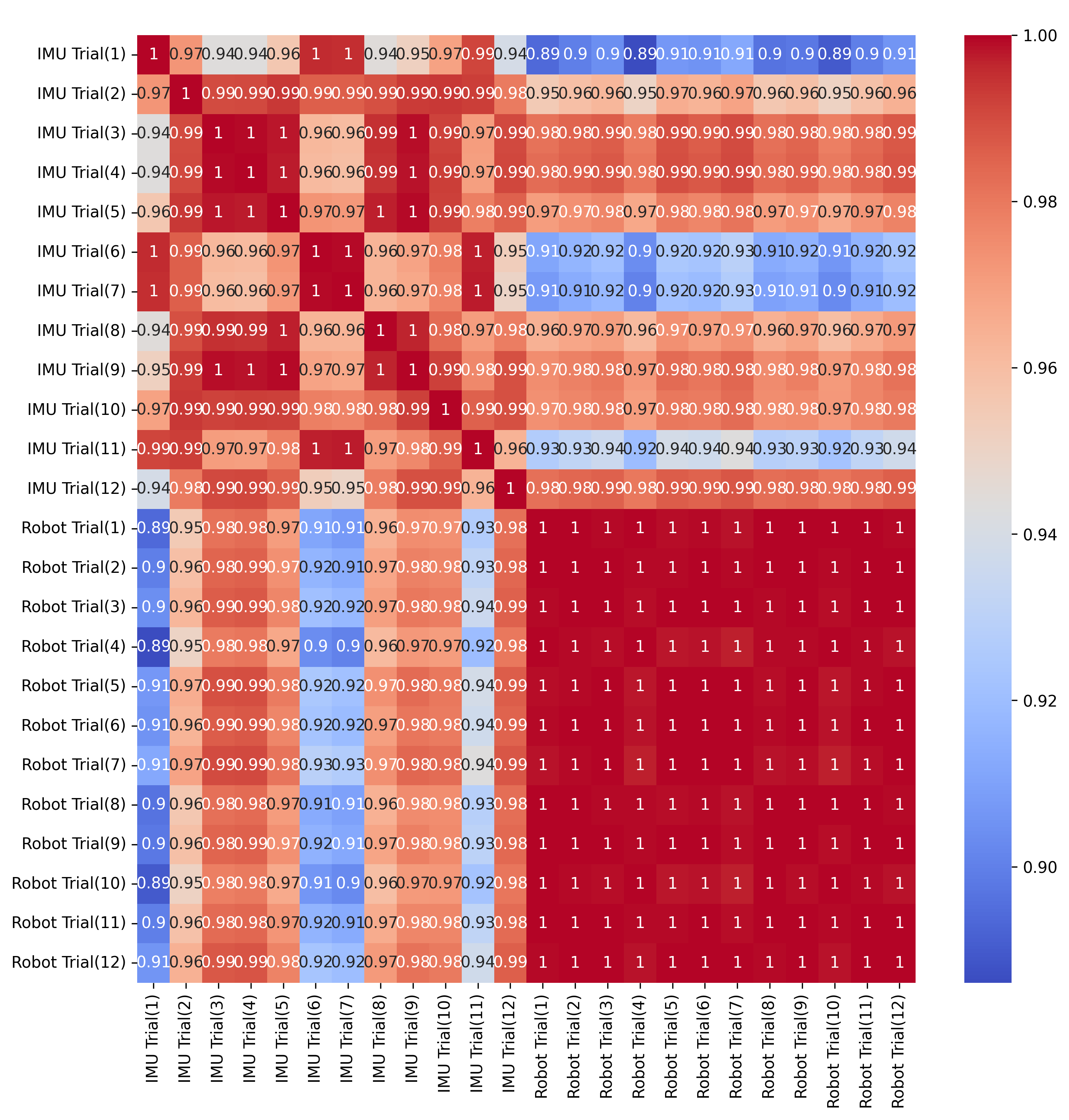

Supplement: Supplementary file 1 [file sensors-25-00002-s001.zip › Supplementary Materials/S7.png]

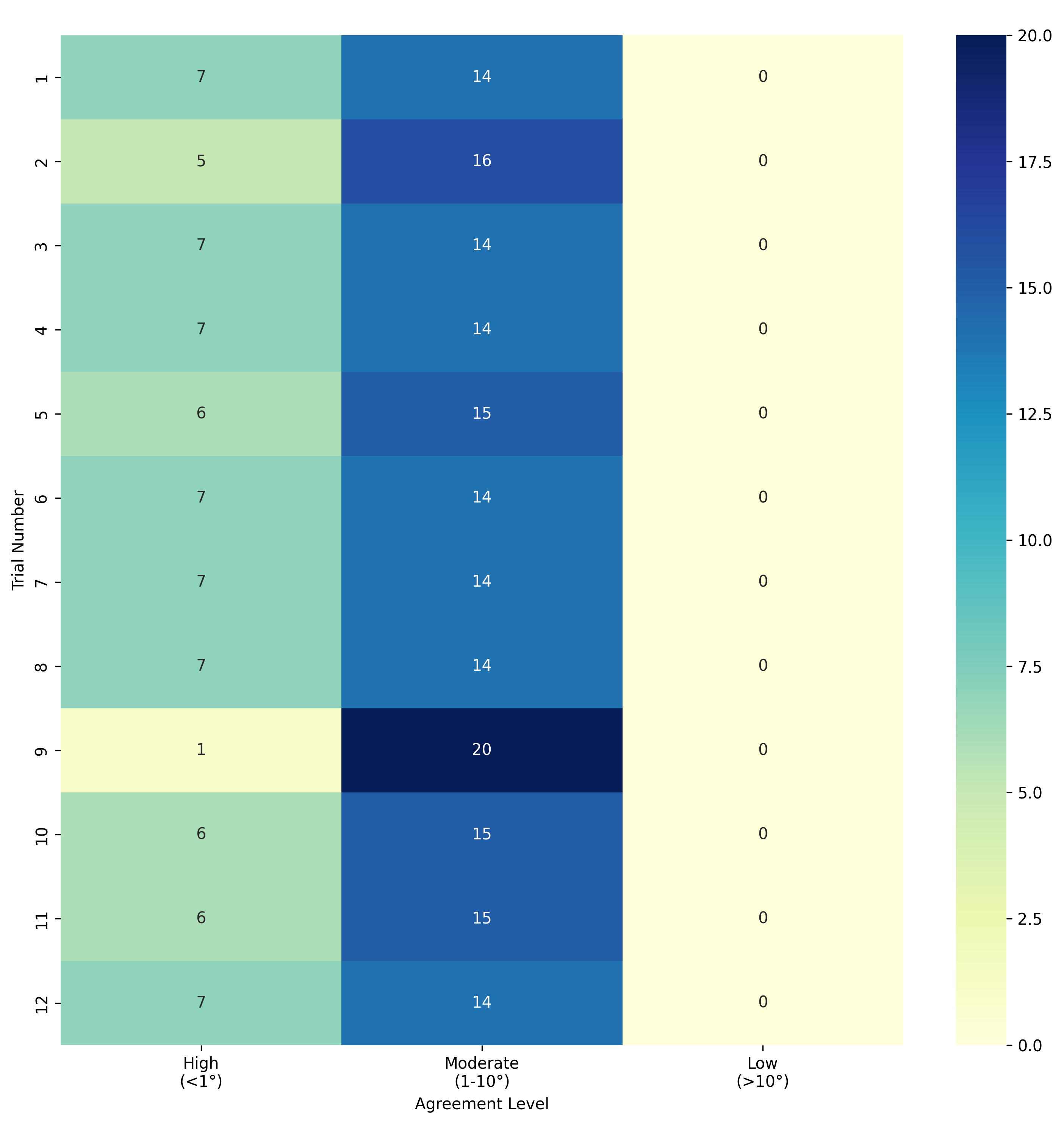

Supplement: Supplementary file 1 [file sensors-25-00002-s001.zip › Supplementary Materials/S8.png]

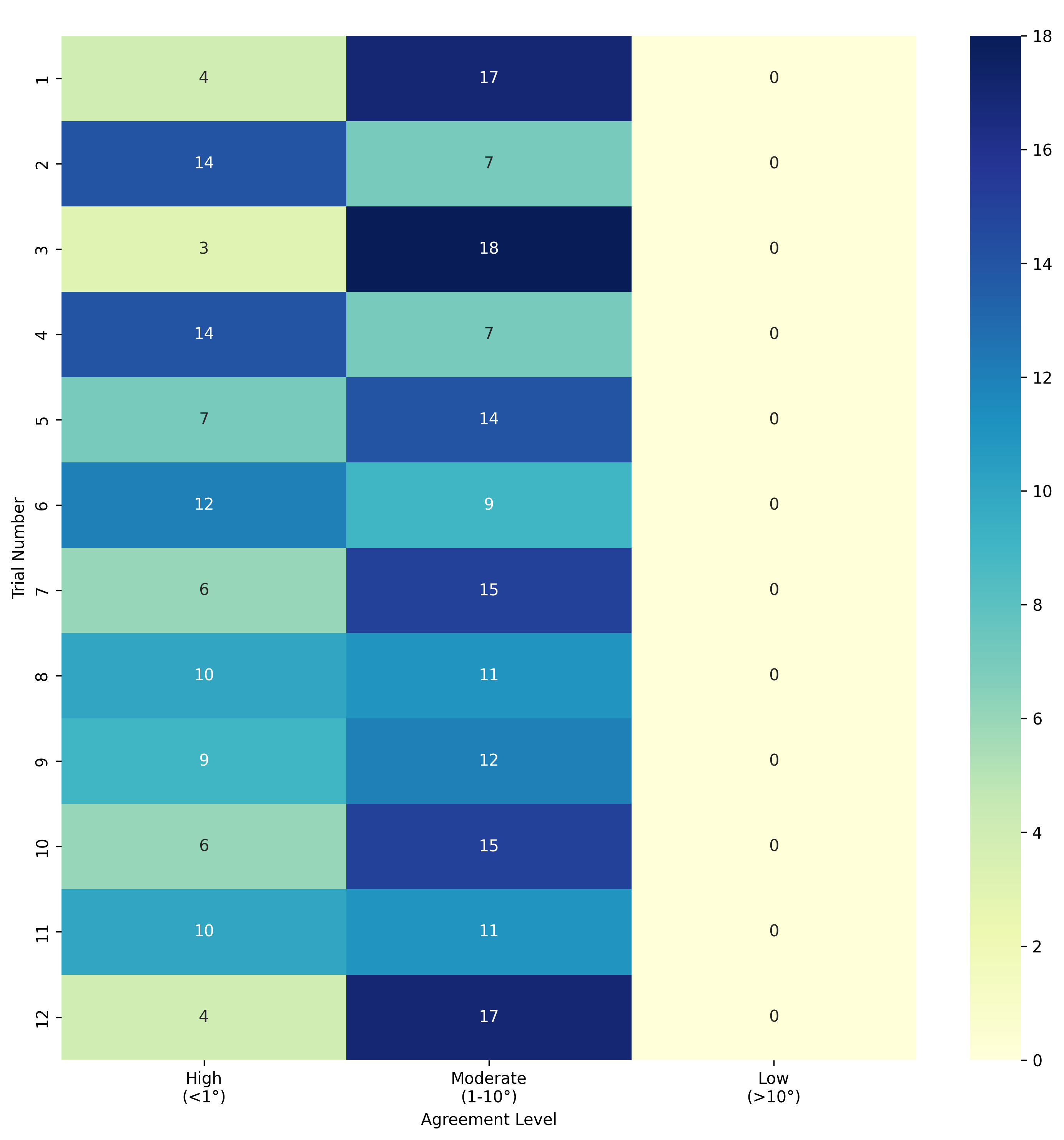

Supplement: Supplementary file 1 [file sensors-25-00002-s001.zip › Supplementary Materials/S9.png]
